# Double-multilayer monochromators for high-energy and large-field X-ray imaging applications with intense pink beams at SPring-8 BL20B2

**DOI:** 10.1107/S1600577522006610

**Published:** 2022-07-15

**Authors:** Takahisa Koyama, Yasunori Senba, Hiroshi Yamazaki, Tomoyuki Takeuchi, Masayuki Tanaka, Yasuhiro Shimizu, Koji Tsubota, Yasuhisa Matsuzaki, Hikaru Kishimoto, Takanori Miura, Satsuki Shimizu, Takamitsu Saito, Hirokatsu Yumoto, Kentaro Uesugi, Masato Hoshino, Jumpei Yamada, Taito Osaka, Michihiro Sugahara, Nobuteru Nariyama, Yasuhide Ishizawa, Hiroko Nakano, Choji Saji, Kyo Nakajima, Koji Motomura, Yasumasa Joti, Makina Yabashi, Haruhiko Ohashi

**Affiliations:** a Japan Synchrotron Radiation Research Institute (JASRI), 1-1-1 Kouto, Sayo, Hyogo 679-5198, Japan; b RIKEN SPring-8 Center, 1-1-1 Kouto, Sayo, Hyogo 679-5148, Japan; Bhabha Atomic Research Centre, India

**Keywords:** double-multilayer monochromator, multilayer mirror, high energy, X-ray imaging, large field of view

## Abstract

In this study, double-multilayer monochromators that generate intense, high-energy, pink X-ray beams are designed, installed and evaluated at the SPring-8 medium-length (215 m) bending-magnet beamline BL20B2 for imaging applications. Two pairs of W/B_4_C multilayer mirrors are designed to utilize photon energies of 110 keV and 40 keV with bandwidths of 0.8% and 4.8%, respectively, which are more than 100 times larger when compared with the Si double-crystal monochromator (DCM) with a bandwidth of less than 0.01%. At an experimental hutch located 210 m away from the source, a large and uniform beam of size 14 mm (V) × 300 mm (H) [21 mm (V) × 300 mm (H)] was generated with a high flux density of 1.6 × 10^9^ photons s^−1^ mm^−2^ (6.9 × 10^10^ photons s^−1^ mm^−2^) at 110 keV (40 keV), which marked a 300 (190) times increase in the photon flux when compared with a DCM with Si 511 (111) diffraction. The intense pink beams facilitate advanced X-ray imaging for large-sized objects such as fossils, rocks, organs and electronic devices with high speed and high spatial resolution.

## Abstract 2

Double-multilayer monochromators for high-energy and large-field X-ray imaging applications are designed, installed and evaluated at the SPring-8 medium-length (215 m) bending-magnet beamline BL20B2.

## 1. Introduction

X-ray imaging is one of the most significant applications of brilliant synchrotron radiation sources. In particular, a long X-ray beamline, in which the distance between the source and the experimental station is typically greater than 100 m, facilitates imaging analyses of large objects (over 10 cm). In such cases, the objects tend to be relatively thick, and a high-energy X-ray beam with high transmissivity is required to perform computed tomography. For example, the typical penetration depths (that is, 1/e attenuation length) at X-ray energy 100 keV for water, limestone and iron are 60 mm, 20 mm and 3.4 mm, respectively.

To guide high-energy X-rays to the experimental stations, a double-crystal monochromator (DCM) with Si perfect crystals is widely utilized as the primary beamline monochromator. However, the resolution (Δ*E*/*E*) of the Si DCM is typically below 10^−4^, which is unnecessarily high for most imaging applications. A more relaxed resolution with enhanced photon flux would be more suitable. For example, filtered white beams are utilized to generate intense high-energy X-rays above 30 keV from a bending-magnet or wiggler source (Di Michiel *et al.*, 2005 ▸; Hoshino *et al.*, 2017 ▸; Stevenson *et al.*, 2017 ▸; Mittone *et al.*, 2020 ▸). At SPring-8 beamline BL28B2, X-rays from a bending-magnet source were filtered using a 0.5 mm-thick tungsten plate and a 2 mm-thick lead plate to achieve a flux density of approximately 10^11^ photons s^−1^ mm^−2^ with a peak energy of 200 keV and an energy width of 100 keV (Δ*E*/*E* = 0.5) (Hoshino *et al.*, 2017 ▸). Although the flux of the filtered white beam is very high, a broad spectrum of the white beam inevitably generates artifacts in absorption imaging owing to the variations in penetration depth. This effect, known as beam hardening, hinders accurate analysis. To produce a modest-resolution X-ray beam while suppressing the beam-hardening effect, the use of a double-multilayer monochromator (DMM) (Chu *et al.*, 2002 ▸; Stampanoni *et al.*, 2007 ▸; Rack *et al.*, 2008 ▸, 2009 ▸; Kastengren *et al.*, 2012 ▸; Wilde *et al.*, 2016 ▸; Weitkamp *et al.*, 2017 ▸; Mittone *et al.*, 2020 ▸) is promising. One can design a multilayer with a medium resolution of 10^−2^ even for high-energy (approximately 100 keV) X-rays. A drastic increase in the photon flux, when compared with the performance of the Si DCM, is expected when ‘pink’ beam generated by the DMM is used.

However, there are some challenges in the production and utilization of DMMs. Technological limitations have caused the minimum period of the multilayer to be set to a few nanometres, which corresponds to an incident angle of the order of milliradians or less for hard X-rays above 30 keV based on Bragg’s law. Therefore, a long substrate, typically approximately 1 m in length, is needed to accept an incident beam of the size of a few millimetres. It is difficult to manufacture high-quality multilayer mirrors for long substrates. In addition, it is difficult to maintain a constant height of the exit beam generated by the DMM over a wide range of photon energies without a large translation of the optic along the optical axis. Practically, a specific photon energy may be selected for a single set of DMMs.

In this paper, we report the development, installation and evaluation of DMMs at the SPring-8 BL20B2. This beamline, equipped with a Si DCM, has been utilized mostly for imaging applications since 1999. In 2019, we decided to install two sets of DMMs to produce intense pink beams at photon energies of 40 keV and 110 keV, whereas we retained the DCM to cover a wide photon energy range of 4.4–113 keV with a monochromatic beam. Following the installation of the DMMs, the performance was precisely evaluated in 2021.

The remainder of this paper is organized as follows. In Section 2, we describe the beamline layout, design of the DMMs, and calculation of the heat load on the first mirror of the DMM. In Section 3, we present the evaluation results for the spatial profile and discuss the energy spectrum, flux density, heat load property and stability of the pink beams generated by the DMM. Finally, we summarize the paper in Section 4.

## 2. Design

### 2.1. Beamline layout

**Figure 1:**
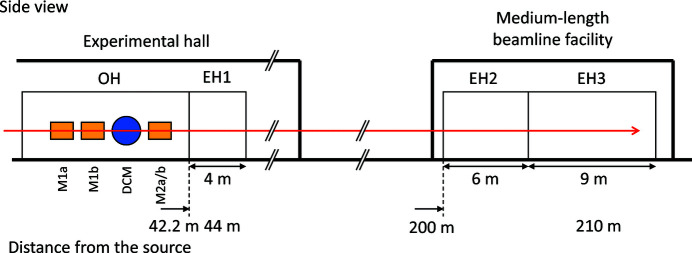
Schematic of the beamline with the optics hutch (OH) and the experimental hutches (EH1, EH2 and EH3). The main optical components, that is, the multilayer mirrors (M1a, M1b and M2a/b) and DCM, are located in the OH.

**Figure 2:**
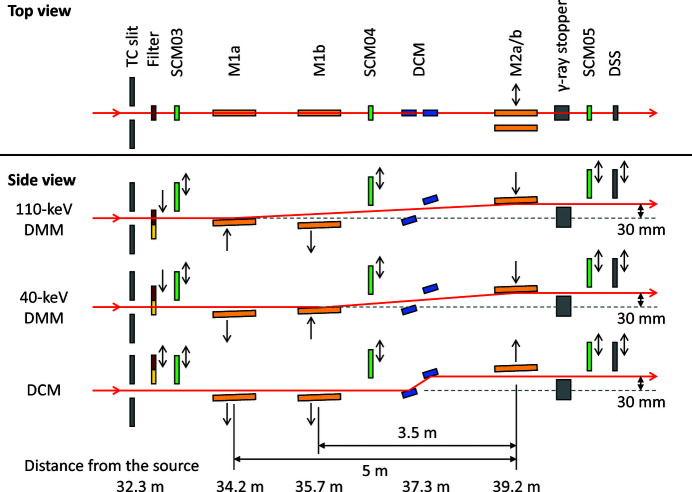
Schematics of the optical configurations for the 110 keV DMM (M1a–M2a), 40 keV DMM (M1b–M2b) and DCM modes. TC slit: transport channel slit; SCM: screen monitor; DCM: double-crystal monochromator; DSS: downstream shutter.

The SPring-8 BL20B2 was constructed and opened to users in 1999 (Goto *et al.*, 2001 ▸). It has an optics hutch (OH) and experimental hutches (EH1, EH2 and EH3), as illustrated in Fig. 1 ▸. OH and EH1 are located in the experimental hall of the storage ring building, whereas EH2 and EH3 are located at the medium-length beamline facility, connected to EH1 through a 150 m-long vacuum pipe of diameter 400 mm. In this study, the optical components in the OH were reassembled. In addition to the existing DCM, DMM chambers consisting of multilayer mirrors M1a, M1b and M2a/b were installed. Fig. 2 ▸ presents a schematic of the optical configurations in the OH. A multilayer mirror pair of M1a and M2a (M1b and M2b) was used to produce a 110 keV (40 keV) X-ray beam, as described in Section 2.2. Filters were used to remove the total-reflection components reflected from the DMMs and reduce the heat load on the first multilayer mirrors (M1a and M1b), as described in Section 2.3.

### 2.2. Design of the double-multilayer monochromator

The optical parameters of the multilayer mirrors are listed in Table 1 ▸. Because the minimum available multilayer period is approximately 1.5 nm, the grazing angle is a few milliradians at approximately 100 keV. In this study, at 110 keV (40 keV), we set the grazing angle to 3 mrad (4.29 mrad) using multilayers with a period of 1.908 nm (3.85 nm). The distance between M1a and M2a (M1b and M2b) of the DMM was set to be 5 m (3.5 m) at 110 keV (40 keV), as depicted in Fig. 2 ▸, so that the offset of the exit beam of the DMM is in accord with that of the DCM (30 mm vertically).

We adopted W/B_4_C multilayers that have good thermal stability even under high heat load conditions (Yanagihara *et al.*, 1993 ▸; Ziegler, 1995 ▸; Takenaka *et al.*, 1996 ▸). Furthermore, the W/B_4_C multilayers have a high electron density contrast, resulting in a high reflectivity from each layer and a smaller number of reflection layers; thus, a wider bandwidth can be expected. Here, the bandwidth means the full width at half-maximum (FWHM) of the reflection profile (Underwood & Barbee, 1981 ▸). The number of periods was set to 200 (50) at 110 keV (40 keV) to achieve a saturated peak reflectivity. The thickness ratio between the W and B_4_C layers was chosen to be 0.5 to suppress the second Bragg peak reflection.

The substrate size and effective area were designed to be 820 mm long × 80 mm wide × 60 mm thick and 800 mm long × 60 mm wide, respectively. This area is large enough to accept the vertical beam size (3σ) of 2.4 mm (3.7 mm) at 110 keV (40 keV) with a grazing angle of 3 mrad (4.29 mrad) and to cover the 58.8 mm horizontal beam at M2a/b (39.2 m from the source). The mirror substrates, made of silicon, were polished by JTEC Corporation. The figure errors of each mirror substrate were found to be 1.7–1.9 nm peak-to-valley by measuring the stitching interferometry in full length of the effective area. Surface roughness values were found to be 0.16–0.18 nm RMS by measuring with a white-light interferometer with 50× objective lens.

**Figure 3:**
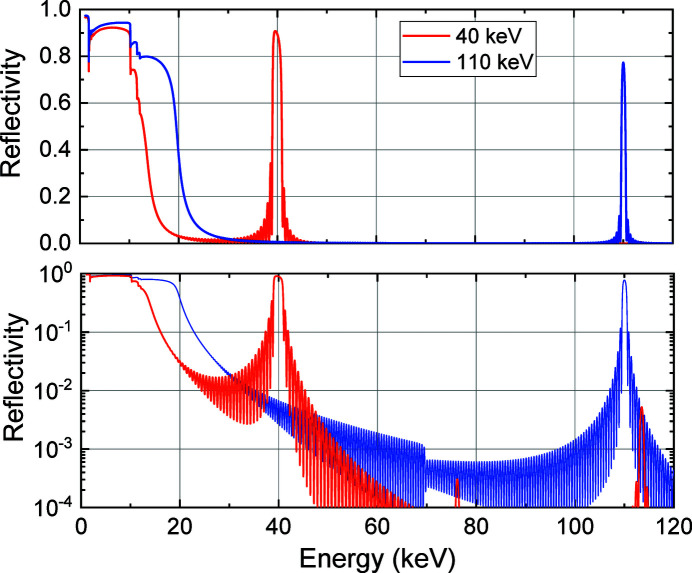
Calculated multilayer reflectivities (single reflection) plotted as functions of the X-ray energy for the 40 keV and 110 keV multilayer mirrors. The vertical axes of the graphs are drawn in linear (upper) and log (lower) scales.

The calculated multilayer reflectivities (single reflection) are plotted as functions of the X-ray energy for the 110 keV and 40 keV multilayer mirrors in Fig. 3 ▸. These calculations used Parratt’s recurrence formula (Parratt, 1954 ▸) and optical constants from the NIST database (Chantler *et al.*, 2005 ▸). A peak reflectivity of 0.77 (0.91) with a bandwidth of 0.8% (4.8%) was obtained for the 110 keV (40 keV) multilayer mirror, for which the multilayer roughness was set to be 0.4 nm RMS. Under these conditions, we can expect an increase in the flux of more than two orders of magnitude when compared with the Si DCM with narrow bandwidths of 1.5 × 10^−4^, 2.7 × 10^−5^ and 1 × 10^−5^ for the Si 111, 311 and 511 reflections, respectively. It should be noted that low-energy X-rays below the critical energy of 20 keV (15 keV) for the 110 keV (40 keV) DMM are totally reflected and removed by filters, as described in Section 2.3.

We set the maximum difference in the multilayer periods between the mirror pair to be ±0.5% to suppress the deviation in the grazing angles. For example, in the case of a grazing angle of 3 mrad, a difference of 0.5% leads to a displacement of 5 mm at EH3 located at 160–170 m from the DMM. This displacement can be corrected by translating the imaging system. The tolerance of the in-plane coating uniformity (that is, the multilayer period error in various positions) was set to be ±0.2% to suppress the deviation of the diffracted photon energies and maintain the reflectivity of the double reflection at as high as 50% when compared with that of the ideal case. The W/B_4_C multilayers were coated by Rigaku Innovative Technologies, Inc.

**Figure 4:**
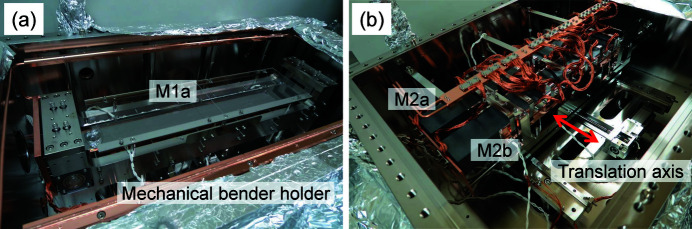
Photographs of the insides of the M1a (*a*) and M2a/b (*b*) chambers.

For the mechanical design, a mirror holder was mounted on a solid table placed on three vertical translation stages to enable variations in the vertical, pitch and roll positions. These stages and the solid table were placed outside the vacuum chamber. The solid table supports the mirror holder placed in the vacuum chamber with pillars and bellows. M1a and M1b were mounted on a mechanical bender holder to correct the deformation of the mirror shape caused by the self-weight and possible heat load, as shown in Fig. 4 ▸(*a*). Both ends of the mirror were clamped by box-shaped rotary clamps. The rotary clamps were connected to a curved rod placed under the mirror, and, by bending this rod into an arch shape, a rotary moment was given to the clamp, resulting in bending the mirror to a cylindrical surface. Both sides of the first mirror (M1a and M1b) substrates were clamped with copper blocks that were cooled by water flowing in cooling pipes. The second mirror (M2a and M2b) substrates were mounted in a similar manner but cooled with a flexible heat mesh. M2a and M2b were placed in parallel and moved horizontally through a translation stage to switch them, as shown in Fig. 4 ▸(*b*). These mechanics were designed and manufactured by TOYAMA Co., Ltd.

It should be noted that the three optical configurations (that is, 40 keV DMM, 110 keV DMM and DCM) can easily be switched through a simple movement of the optical elements in the vacuum chambers within approximately 10 min.

### 2.3. Design of filters

**Figure 5:**
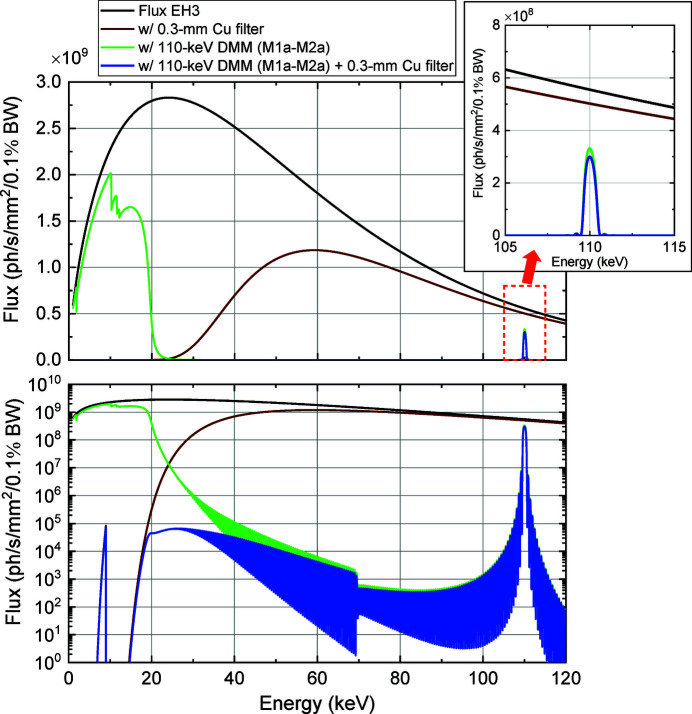
Calculated photon flux densities at EH3 under various conditions. The black line represents the photon flux density generated by the source. The green and brown lines represent the densities obtained using the 110 keV DMM (M1a–M2a) and 0.3 mm Cu filter, respectively. The blue line represents the density obtained using both the 110 keV DMM and 0.3 mm Cu filter. The inset depicts an enlarged representation of the plot enclosed in the dashed square. The vertical axes of the graphs are drawn in linear (upper) and log (lower) scales.

Filters were installed upstream of the M1a chamber to reduce the total-reflection components reflected from the DMMs and suppress the heat load on the first multilayer mirrors (M1a and M1b). Based on the thermal conductivity and transmissivity, the filter materials and thicknesses were chosen to be Cu, 0.3 mm for 110 keV DMM, and SiC, 2 mm for 40 keV DMM. The filter plates were mounted on a water-cooled holder. Figs. 5 ▸ and 6 ▸ depict the calculated flux densities at EH3 with or without the filters and DMMs. Tables 2 ▸ and 3 ▸ summarize the filter transmissions and amounts of power absorbed by the filters, M1a and M1b. It was confirmed that the total-reflection components were effectively reduced by using the filters while maintaining the transmission at 110 keV to be 0.9 and that at 40 keV to be 0.7. In addition, the filter is effective for reducing the unwanted heat load on M1a and M1b to suppress possible thermal deformation. The maximum irradiation power incident on M1a (M1b) of 156 W (195 W) was reduced to 56 W (100 W) using a 0.3 mm Cu (2 mm SiC) filter, which corresponds to a power of 55 W (95 W) absorbed by M1a (M1b). It should be noted that the degradation of the image quality was negligible, even with the filters.

## 3. Results of characterization

### 3.1. Spatial profiles

We characterized the spatial profiles of the beams reflected from the DMMs at EH3 using an X-ray imaging detector, which consists of an imaging unit (AA60, Hamamatsu Photonics KK), a phosphor screen [10 µm-thick P43 (Gd_2_O_2_S:Tb^+^)], an sCMOS camera (ORCA Flash4.0, Hamamatsu Photonics KK) and a tandem lens with *f* = 105 mm and 50 mm. The effective pixel size was 13.13 µm with a field of view of the size of a 27 mm square.

**Figure 7:**
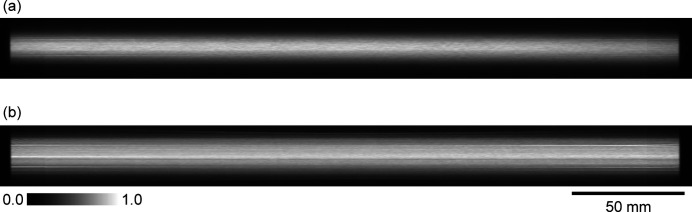
Beam profiles of the (*a*) 110 keV DMM at EH3 and (*b*) 40 keV DMM at EH3.

The images obtained using the 110 keV and 40 keV DMM are presented in Figs. 7 ▸(*a*) and 7 ▸(*b*), respectively. To cover the whole beam image, the detector was moved in steps of 20 mm in the horizontal direction, and 15 images were merged to compose a single picture. The intensity fluctuation in the horizontal direction, which represents the standard deviation of the average intensity at the center part of each image, was 6.2% for the 110 keV DMM and 1.7% for the 40 keV DMM. Therefore, an almost uniform intensity distribution was obtained over the entire horizontal direction. However, a vertical intensity fluctuation appeared in the beams (shown in Fig. 7 ▸ as horizontal white stripes) owing to the figure error on the mirror surface. The contrast of these intensity fluctuations can be reduced using a diffuser.

The beam sizes were measured from the observed images, as summarized in Table 4 ▸, which agreed with the values calculated using the beam divergence.

### 3.2. Energy spectra

**Figure 8:**
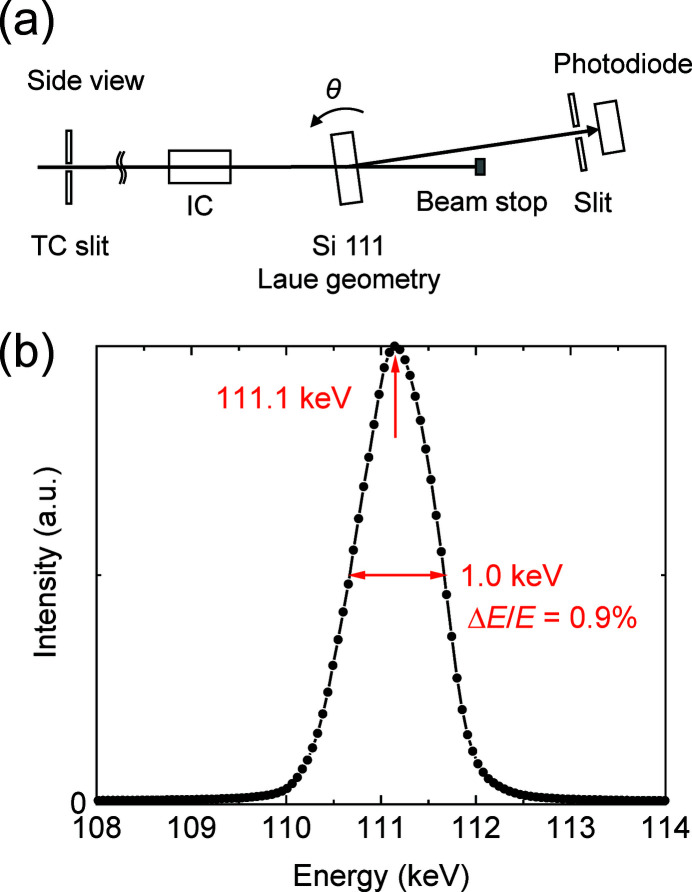
(*a*) Schematic of the spectrum measurement for the 110 keV DMM and (*b*) the measured spectrum.

We evaluated the energy spectra of the beams reflected from the DMMs at EH1. Fig. 8 ▸(*a*) depicts a schematic of the 110 keV DMM spectrum measurement. The aperture of the transport channel (TC) slit, placed in the OH, was set to be 1.0 mm (V) × 5.0 mm (H). The θ–2θ scan was performed using Si 111 diffraction in Laue geometry with a thickness of 10 mm. The measured spectrum was plotted as a function of the X-ray energy, as depicted in Fig. 8 ▸(*b*). The measured peak energy and energy width were 111.1 keV and 1.0 keV, respectively, which correspond to an energy resolution (Δ*E*/*E*) of 0.9%. This value was consistent with the designed value of 0.8%.

**Figure 9:**
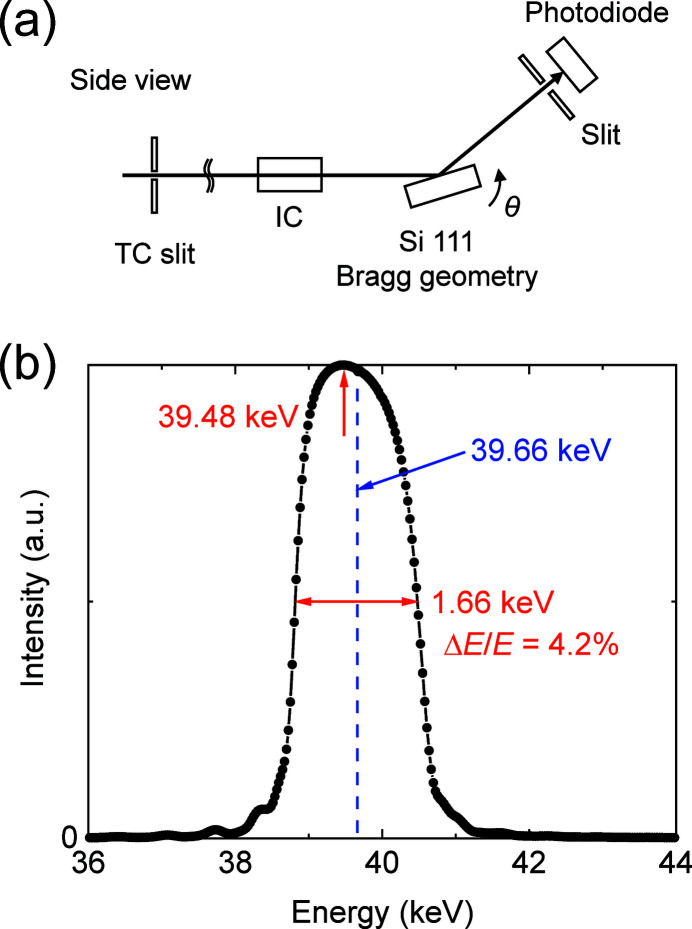
(*a*) Schematic of the spectrum measurement for the 40 keV DMM and (*b*) the measured spectrum.

The spectrum of the beam reflected from the 40 keV DMM was measured in a similar manner, except for the configuration of the analyser crystal in Bragg geometry, as depicted in Fig. 9 ▸(*a*). The measured peak energy, energy width and energy resolution (Δ*E*/*E*) were 39.48 keV, 1.66 keV and 4.2%, respectively. The final value was almost consistent with the designed value of 4.8%.

### 3.3. Flux density

The flux densities at EH1 and EH3 were measured using spatial apertures and an Si-PIN photodiode (s14537-320, Hamamatsu Photonics KK) in combination with a conversion relationship from the photodiode output to the photon flux, as reported in the literature (Nariyama *et al.*, 2004 ▸). The measured flux densities are summarized in Table 5 ▸, with the values calculated using *SPECTRA* software (Tanaka & Kitamura, 2001 ▸). We obtained reasonably high flux densities: 70%–90% of the calculated values. In contrast, the flux density at 110 keV (40 keV) using the DCM with Si 511 (111) diffraction at EH3 was calculated to be 5.5 × 10^6^ photons s^−1^ mm^−2^ (3.6 × 10^8^ photons s^−1^ mm^−2^). Therefore, the flux density generated by the 110 keV (40 keV) DMM at EH3 was increased by a factor of 300 (190) when compared with the calculated value of the DCM.

### 3.4. Heat load

**Figure 10:**
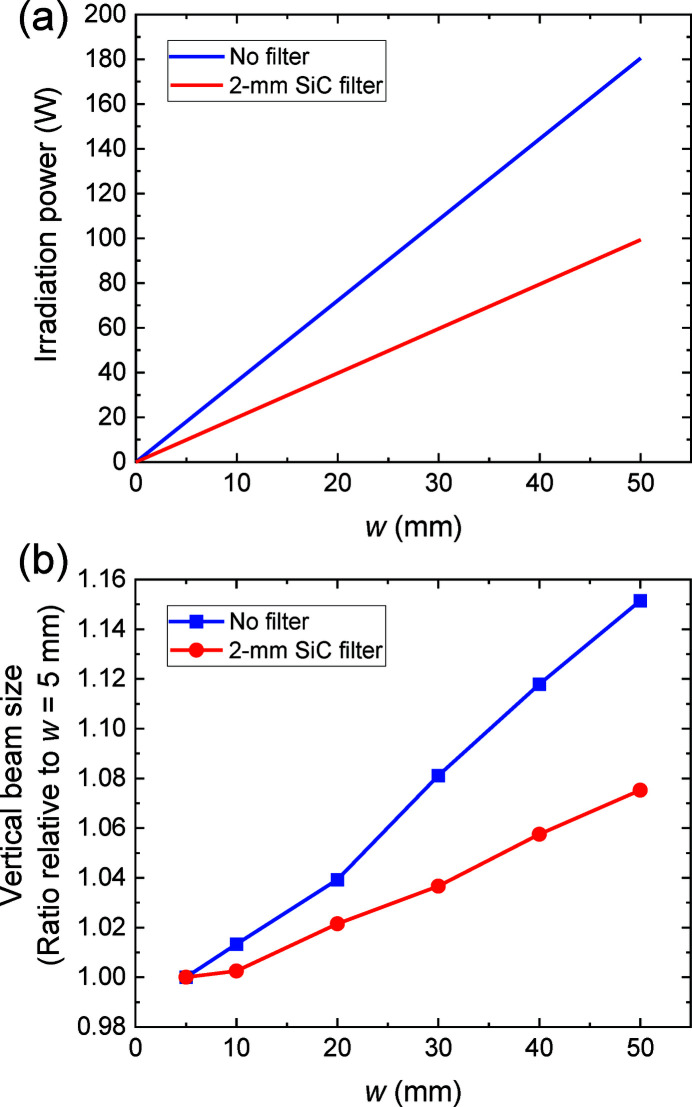
(*a*) Calculated power incident on M1b plotted as a function of the TC slit width (*w*). The blue (red) line indicates the incident power without a filter (with a 2 mm SiC filter). (*b*) Vertical beam size at EH3 as a function of *w*. The size is normalized by the beam size at *w* = 5 mm. The blue (red) symbols represent the beam size without a filter (with a 2 mm SiC filter).

We evaluated the heat load on the mirrors by varying the horizontal width of the TC slit. We also investigated the effectiveness of the filters. The radiation power incident on the first mirror (M1b) of the 40 keV DMM was changed from 10 W to 99 W using the 2 mm SiC filter and from 18 W to 181 W without the filter, as depicted in Fig. 10 ▸(*a*). The beam images were observed at EH3. We observed an increase in the vertical beam size with respect to the opening of the slit width, as depicted in Fig. 10 ▸(*b*). This was caused by the deformation of the mirror surface to a convex shape owing to the increased heat load. We found that the deformation was reduced by using the filter, as expected.

We evaluated quantities of the thermal deformation (curvature) of the mirror under the assumption that the mirror is flat with zero heat load and the deformation is cylindrical in the first approximation. By using an approximate formula of a cylinder focusing mirror (that is, the relationship among a meridional radius of curvature, a source to mirror distance, a mirror to focal point distance, and a glancing angle), the thermal deformation can be evaluated from the vertical beam size increase and the optical layout. The vertical beam size increase was observed to be 15% (7.5%) from Fig. 10 ▸(*b*), thus we evaluated the radius of curvature of the M1b mirror to be 91 km (184 km) and the thermal deformation to be 0.92 µm (0.45 µm) with a convex shape, when varying the TC slit width (*w*) from 5 to 50 mm without filter (with 2 mm SiC filter). We also evaluated the thermal deformation using thermal-structural analysis by finite-element method simulation, in terms of the power absorbed by the substrate taking into consideration the filter transmission and the multilayer reflectivity. The evaluated thermal deformation was 0.90 µm (0.45 µm) with a convex shape when varying *w* from 5 to 50 mm without filter (with 2 mm SiC filter). These values were consistent with ones evaluated from the beam size increase.

In these measurements, the temperatures of the mirror substrates were monitored by thermocouple sensors placed on the side end of the mirror surface. The temperature of the filter holder was also monitored. The measured temperature of M1b (the filter holder) was maintained at approximately 33°C (41°C), even under the highest heat load condition, that is, at a slit width of 50 mm without (with) the filter.

### 3.5. Stability

**Figure 11:**
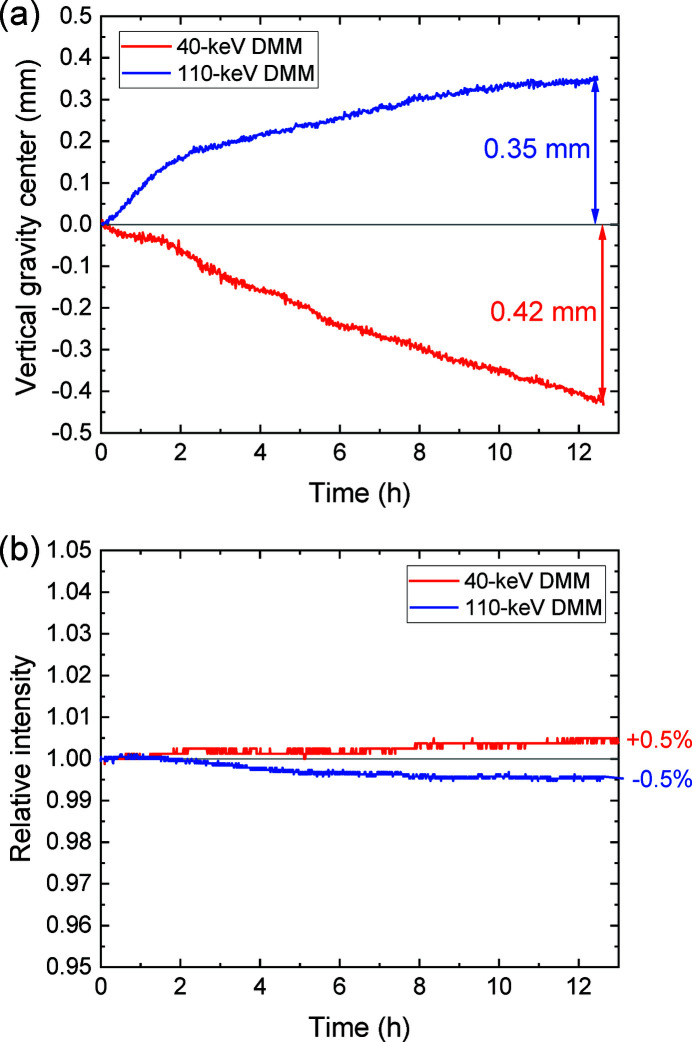
Result of stability measurements for the 40 keV and 110 keV DMM. (*a*) Vertical image gravity centers plotted as a function of time. (*b*) Variations in relative intensity plotted as a function of time.

We evaluated the stability of the DMMs by monitoring the beam positions and fluctuation of the intensities over 12.5 h. The TC slit width was set to be 10 mm, meaning a horizontal beam size of 65 mm at EH3. Beam images were captured at intervals of 1 min at EH3, and the intensity was monitored using an ionization chamber at EH1. Fig. 11 ▸ presents the measured results. The gravity center of the intensity profile in the vertical direction drifted 350 µm upward (420 µm downward) gradually for the 110 keV (40 keV) DMM, as depicted in Fig. 11 ▸(*a*). These drift values, which were measured to be approximately 2% of the vertical beam size, corresponded to angular drifts as low as approximately 1 µrad. It should be noted that in these measurements the temperature variations (measured upstream and downstream of the granite tables for M1a, M1b and M2a/b) were less than 0.06°C. The relative intensity drifts, which were as small as 0.5%, were plotted as functions of time, as presented in Fig. 11 ▸(*b*). The cause of the intensity drifts could be angular drift of the mirrors. One possible reason for the angular drift might be a gradual heating of the M1 mirror holder due to beam scattering. The heat could be transmitted to the solid table and the translation stages, resulting in the angular displacement.

## 4. Summary

We have designed, installed and evaluated 110 keV and 40 keV double-multilayer monochromators (DMMs) to produce intense, high-energy, pink X-ray beams at SPring-8 BL20B2. Two pairs of W/B_4_C multilayer mirrors were designed to utilize the photon energies of 110 keV and 40 keV with bandwidths of 0.8% and 4.8%, respectively, which are more than 100 times larger when compared with the Si DCM with a bandwidth of less than 0.01%. We characterized the beam properties and reported the following. At EH3, located 210 m from the source, a large and uniform beam of size 14 mm (V) × 300 mm (H) [21 mm (V) × 300 mm (H)] was generated at 110 keV (40 keV). The energy spectrum measurements indicated that the measured energy and bandwidth were almost consistent with the designed values. The measured flux densities at EH3 were 1.6 × 10^9^ photons s^−1^ mm^−2^ (6.9 × 10^10^ photons s^−1^ mm^−2^) at 110 keV (40 keV), which indicated a 300 (190) times increase in the photon flux when compared with the DCM with Si 511 (111) diffraction. The filters used in this study were effective in reducing the total-reflection components reflected from the DMMs and suppressing the heat load on the first multilayer mirrors. The result of the 12.5 h stability measurements indicated that the beam positions observed at EH3 drifted slightly, by approximately 2% of the vertical beam size, which corresponded to angular drifts as low as approximately 1 µrad. The relative intensity variations were as small as 0.5%.

The intense pink beams generated by the installed DMMs facilitate advanced X-ray imaging for large objects such as fossils, rocks, organs and electronic devices with high speed and high spatial resolution.

## Figures and Tables

**Figure 6:**
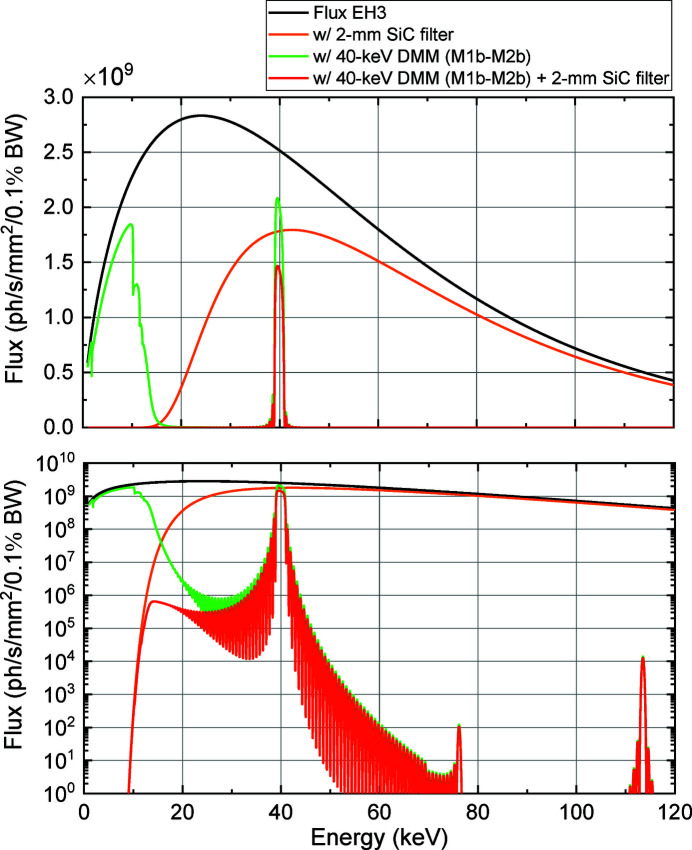
Calculated photon flux densities at EH3 under various conditions. The black line represents the photon flux density generated by the source. The green and orange lines represent the densities obtained using the 40 keV DMM (M1b–M2b) and 2 mm SiC filter, respectively. The red line represents the density obtained using both the 40 keV DMM and 2 mm SiC filter. The vertical axes of the graphs are drawn in linear (upper) and log (lower) scales.

**Table 1:** Design parameters of the multilayer mirrors

Working photon energy	110 keV	40 keV
Name	M1a, M2a	M1b, M2b
Substrate material	Si	
Substrate size	820 mm long × 80 mm wide × 60 mm thick	
Effective area	800 mm long × 60 mm wide	
Coating	W/B_4_C multilayer	
Multilayer period (*d*)	1.908 nm	3.85 nm
Period matching between mirrors	±0.5%	
Coating uniformity	±0.2%	
Gamma (W-layer thickness/*d*)	0.5	
Number of periods	200	50
Bragg angle	3 mrad	4.29 mrad
Peak reflectivity	0.77	0.91
Energy resolution (Δ*E*/*E*)	0.8%	4.8%
Effective roughness	0.4 nm RMS	0.4 nm RMS

**Table 2:** Filter transmissions and absorbed powers for the 110 keV DMM with an incident power of 156 W

Filter	0.3 mm Cu
Transmission	
20 keV	1.1 × 10^−4^
110 keV	0.90
Absorbed power	
Filter	100 W
M1a	55 W†

† 123 W without filter.

**Table 3:** Filter transmissions and absorbed powers for the 40 keV DMM with an incident power of 195 W

Filter	2 mm SiC
Transmission	
15 keV	8.6 × 10^−3^
40 keV	0.71
Absorbed power	
Filter	95 W
M1b	95 W†

† 160 W without filter.

**Table 4:** Measured beam sizes of the 110 keV and 40 keV DMMs

	110 keV	40 keV
EH1	2.7 mm (V) × 62.6 mm (H)	4.0 mm (V) × 62.6 mm (H)
EH3	14.4 mm (V) × 297 mm (H)	20.8 mm (V) × 297 mm (H)

**Table 5:** Measured (calculated) flux densities at 110 keV and 40 keV

	110 keV	40 keV
EH1	3.9 × 10^10^ (5.2 × 10^10^) photons s^−1^ mm^−2^	1.3 × 10^12^ (1.6 × 10^12^) photons s^−1^ mm^−2^
EH3	1.6 × 10^9^ (2.4 × 10^9^) photons s^−1^ mm^−2^	6.9 × 10^10^ (7.1 × 10^10^) photons s^−1^ mm^−2^
